# Comparison of different methods used in drugs of abuse for sample validity testing including pH methods, specific gravity methods, TECO™ Drug Adulteration Test Strip and oxidant assay

**DOI:** 10.1515/almed-2021-0026

**Published:** 2021-06-04

**Authors:** Ashraf Mina, John Stathopoulos, Taveet Sinanian, Leah McNeice, Deirdre Holmes, Kristi-Lee Fletcher, Emily Bottero, Shanmugam Banukumar, Santiago Vazquez

**Affiliations:** NSW Health Pathology, Forensic & Analytical Science Service (FASS), Toxicology Unit, Macquarie Hospital, Sydney, NSW, Australia; Faculty of Medicine and Health, Sydney University, Sydney, NSW, Australia

**Keywords:** drug adulteration, oxidant assay, pH, sample validity, specific gravity

## Abstract

**Objectives:**

In the absence of sample validity testing, a healthcare provider may fail to identify a patient’s adulteration of their urine sample. This study compared different methods for specific gravity (SG), pH, TECO™ Drug Adulteration Test Strip (dipstick) and oxidant assay to explain the differences and also make an informative decision on method selection.

**Methods:**

Creatinine, SG and pH measurements are essential in sample validity testing. SG and pH automated chemical methods are compared against pH meter method, SG refractometer and dipstick method. Also, oxidant assay was compared against dipstick method.

**Results:**

SG chemical method agreement with refractometer is 81.9% and with dipstick method is 64.7%. The refractometer method agreement with dipstick method is 66.1%. pH chemical method agreement with pH Meter method is 74.3% and with dipstick method is 81.4%. pH meter method agreement is 85.7% with dipstick method. Results were analysed using Deming regression analysis and F-test. SG chemical method correlated better with refractometer than the dipstick method. Oxidant assay correlated well with dipstick method in detecting adulterants such as pyridinium chlorochromate, nitrite and bleach.

**Conclusions:**

Varying degrees of differences were seen in the SG and pH measurements. These differences were both method and instrument dependent. The automated chemical methods are recommended alongside oxidant assay for consistency, accuracy and faster turn-around time as part of sample validity testing for drugs of abuse.

## Introduction

Urine drug testing is common for emergency medicine, prescription management, workplace drug testing and the criminal justice system. The definition of illicit drug use is the use of amphetamines, ecstasy, cocaine, benzodiazepines, cannabinoids (marijuana), opiates, heroin, oxycodone, hallucinogens, inhalants, fentanyl, or the non-medical use of prescription psychotherapeutics such as prescription pain relievers, tranquilisers, stimulants and sedatives. Because drug testing is associated with personal, occupational and legal implications, primary care physicians must be confident in their abilities to interpret urine drug assay results to respond duly to optimise clinical outcomes [1]. Some patients try to defeat human urine drug testing either by orally ingesting detoxification or flushing agents, dilution with water and other liquids and/or adulteration with other chemicals such as bleach or nitrite., and substitution with drug-free human urine or synthetic urine [2].

Adherence can be masked by factors such as dilute urine, cleansing products, urine additives, the quantity of drug consumed, time since the last dose, substituted urine sample, synthetic urine substitutes, the laboratory’s assay cut-off levels and measurement uncertainty (MU). Negative results in a dilute urine specimen may lead to misinterpretation of drug use in an individual. It is mandated by the Substance Abuse and Mental Health Services Administration (SAMHSA) in the United States of America that creatinine, specific gravity (SG) and pH must be tested on urine samples to verify specimen validity [3].

Other protocols are recommended when testing for urine adulteration [4], [5], [6]. These protocols include measuring the temperature, creatinine, specific gravity, oxidant assay and pH.

Fresh normal urine should have the following typical characteristics: Temperature between 32.5 and 37.7 °C or 90.5–99.8 °F, pH within 4.7–7.8 [7], [8], specific gravity within a range of 1.003–1.035 g/mL [7], [9] and the creatinine concentration of 80–200 mg/dL (7.07–17.68 mmol/L) [9], [10]. If any of the urine parameters are outside the specified range, there is reason to suspect that the urine sample may have been adulterated.

In this study, the aim is to compare different methods in measuring pH and SG using Beckman–Coulter AU5810 chemistry analyser, pH Meter, SG refractometer and dipstick.

Also, interference from pyridinium chlorochromate, nitrite, nitrate, sodium hypochlorite using oxidant assay was evaluated.

The population of patients that will benefit from the information presented in this article are patients undergoing testing for drugs of abuse or other clinical evaluation where these tests are required. This article evaluated the performance of six different methods of measuring pH and SG and explained the differences. Also evaluated oxidant assay with dipstick. This will allow laboratories to both evaluate the methods in use and realise the variations between different methods and to make an informed decision when they employing these methods.

## Materials and methods

The analysers used are Beckman–Coulter AU5810 chemistry analyser, which was used for testing pH and Specific Gravity (SG). The chemical reagents used on the Beckman–Coulter AU5810 chemistry analyser, are from Thermo Fisher. pH-Detect Kit (CDF100054), pH Calibrator set. (CDF100283), pH 7 Control (CDF100284) and pH 10 Control (CDF100285). Gravity detect kit (CDF1194), gravity-detect low calibrator (CDF1754), gravity-detect high calibrator (CDF1755), gravity-detect level 1 control (CDF1756), gravity-detect level 2 control (CDF1757) and Thermo Fisher oxidant assay (10,009,958).

Mettler Toledo Seven Compact™ pH/Ion meter S220 is used to measure pH, and micro Palm Abbe digital refractometer PA202 is used to measure SG.

To test for interference the following reagents are used certified blank urine from PM separation (88121-CDF-L UTAK), nitrite certified reference material from Choice Analytical, (IC-N-M-100), bleach (Sodium Hypochlorite) 125 g/L from Jasol (2,066,070), and pyridinium chlorochromate 98% from Aldrich (19,014-4). TECO™ Drug Adulteration Test Strip from Techo Diagnostics (CDA700-25) is used to detect creatinine, pH, SG, nitrite, bleach, pyridinium chlorochromate and glutaraldehyde.

Tested urine samples were a mix of hospitals, correctional centres and clinics patients that are tested regularly in our laboratory. For SG, 204 urine samples were tested using Thermo Fisher chemical method on Beckman–Coulter AU5810 chemistry analyser, refractometer and dipstick. For pH, 210 urine samples were tested using Thermo Fisher chemical method on Beckman–Coulter AU5810 chemistry analyser, pH meter and dipstick. The samples were run within one week using different aliquots on different work stations. The results were analysed using Deming weighted regression analysis and F-test. Also, the agreement percentage was calculated between different methods based on the number of results matching either within or outside the reference interval of the assay.

Serial dilutions of nitrite, nitrate, bleach (sodium hypochlorite) and pyridinium chlorochromate were spiked into blank urine to mimic adulterated urine. These serial dilutions were then tested using both the oxidant assay and the dipstick for comparison. Since nitrite is both endogenous in urine and also can be used as an adulterant, it was tested by spiking certified blank urine using laboratory-grade sodium nitrite and also by using nitrite Certified Reference Material (CRM). The certified blank urine was used in the preparation of these serial dilutions on the initial day of preparation. After the initial testing of the prepared serial dilutions, the samples were reanalysed again after 17 days to test for the stability of adulterants in the urine.

This study did not require Ethical Committee approval because it did not contain any studies with human participants or animals. Additionally, sample validity testing is within the normal scope of testing.

## Results

Deming regression uses paired measurements, (*x*_*i*_, *y*_*i*_), measured with errors (*ε*_*i*_ and *δ*_*i*_), where *x*_*i*_ = *X*_*i*_ + *ε*_*i*_ and *y*_*i*_ = *Y*_*i*_ + *δ*_*i*_, to estimate the intercept (*β*_0_), and the slope (*β*_1_), in the equation ${\bar{Y}}_{i}$ = *β*_0_ + *β*_1_ ${\bar{X}}_{i}$ , where ${\bar{X}}_{i}$ and ${\bar{Y}}_{i}$ are estimates of the “true” (or expected) values of *X*_*i*_ and *Y*_*i*_, respectively. Deming regression is often used for method comparison studies in clinical chemistry to look for systematic differences between two measurement methods. Slope shows the proportional bias; often related to calibration differences between methods, while intercept shows constant bias; may be related to calibration or set point issues. F-statistic is a ratio of two variances, or technically, two mean squares to find out if the means between two populations are significantly different. Under the null hypothesis, F-statistic is approximately 1. In order to reject the null hypothesis that the group means are equal, we need a high F-value.

**Figure 1:**
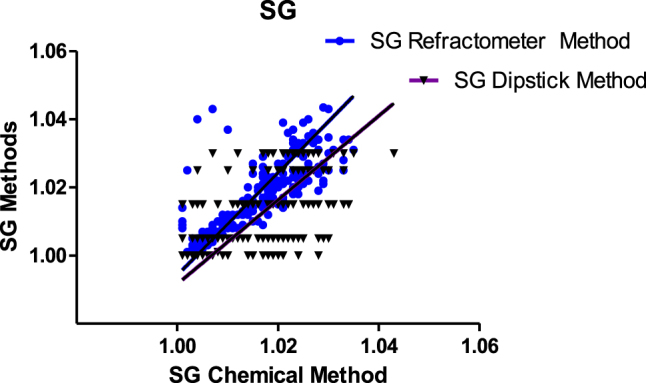
Deming regression analysis for Thermo Fisher SG chemical method vs. refractometer and TECO™ Drug Adulteration Test Strip (dipstick) methods.

The data was analysed using the Deming weighted regression analysis and the F-test (Figure 1 and Table 1). There was a significant difference between the SG refractometer and the SG dipstick methods when compared with the Thermo Fisher chemical method. The SG refractometer method showed both more proportional bias and constant bias than the SG dipstick method when compared to the Thermo Fisher chemical method.

**Table 1:** Deming regression and F-test for: (A) Thermo Fisher SG Chemical method vs. refractometer and SG TECO Drug Adulteration Test Strip (dipstick) methods. (B) Thermo Fisher pH Chemical method vs. pH meter and pH TECO™ Drug Adulteration Test Strip (dipstick) methods.

A) Deming regression analysis	SG Refractometer method	SG TECO Drug Adulteration Test Strip (dipstick)
Equation	${\bar{Y}}_{i}$ = *β*_0_ + *β*_1_ ${\bar{X}}_{i}$	${\bar{Y}}_{i}$ = *β*_0_ + *β*_1_ ${\bar{X}}_{i}$
Intercept (*β*_0_), (95% confidence interval)	−0.5092 (−0.6847 to −0.3337)	−0.2430 (−0.5139–0.02786)
Slope (*β*_1_), (95% confidence intervals)	1.5000 (1.330–1.676)	1.2345 (0.9679–1.501)

**A) F-test**	293.9	83.25
DFn	202.0	202.0
p-Value	<0.0001	<0.0001
Deviation from zero?	Significant	Significant
Total number of values	204	204

**B) Deming regression analysis**	**pH meter method**	**pH TECO™ Drug Adulteration Test Strip (dipstick)**

Equation	${\bar{Y}}_{i}$ = *β*_0_ + *β*_1_ ${\bar{X}}_{i}$	${\bar{Y}}_{i}$ = *β*_0_ + *β*_1_ ${\bar{X}}_{i}$
Intercept (*β*_0_), (95% confidence interval)	0.0666 (−0.5626–0.6959)	−3.582 (−4.710 to −2.454)
Slope (*β*_1_), (95% confidence intervals)	1.0257 (0.9444–1.107)	1.4825 (1.337–1.628)

**B) F-test**	618.2	401.8
DFn	208.0	208.0
p-Value	<0.0001	<0.0001
Deviation from zero?	Significant	Significant
Total member of values	210	210

**Figure 2:**
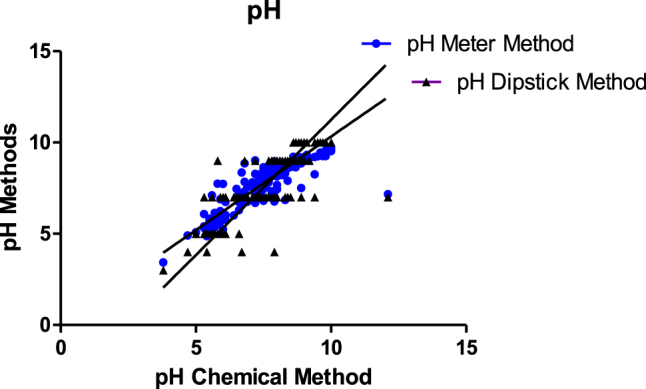
Deming regression analysis for Thermo Fisher pH Chemical method vs. pH meter and pH TECO™ Drug Adulteration Test Strip (dipstick) methods.

There was a significant difference between the pH meter and the dipstick when compared with the Thermo Fisher chemical method (Figure 2 and Table 1). The pH dipstick method showed both more proportional and constant bias than the pH meter method when compared to the Thermo Fisher chemical method.

Also, all the standard solutions used for the calibration of the pH meter were analysed by the Thermo Fisher chemical method on the Beckman–Coulter AU5810 chemistry analyser, and the results matched the concentrations of the pH meter calibrators.

The Techo dipstick is designed to use dry chemistry and gives quantitative results for both pH and SG. The pH is based on the well-known double pH indicator method. SG is based on PKa change of certain pre-treated polyelectrolytes in relation to ionic concentrations.

To measure the precision of Thermo Fisher SG, pH and oxidant methods using the Beckman–Coulter chemistry analyser, control and calibrators samples were tested in replicates of two, twice per day for 20 days. Precision results for SG using micro Palm Abbe digital refractometer and for pH using Mettler Toledo Seven Compact™ pH/Ion meter are summarised in Table 2.

**Table 2:** Precision results for (A) Thermo Fisher pH. (B) Thermo Fisher pH. (C) Thermo Fisher oxidant. (D) SG using micro Palm Abbe digital refractometer. (E) pH using Mettler Toledo Seven Compact™ pH/ion meter.

A) Precision results for Thermo Fisher specific gravity assay (n=80)					
Controls	Control 1	High calibrator		Control 2	
Mean, g/mL	1.014	1.025		1.031	
Within-run SD, g/mL	1.015 ± 0.0004	1.020 ± 0.0002		1.032 ± 0.0002	
Within-run CV, %	0.04	0.05		0.06	
Between-run SD, g/mL	1.015 ± 0.0005	1.020 ± 0.0004		1.032 ± 0.0004	
Between-run CV, %	0.04	0.05		0.06	

**B) Precision results for Thermo Fisher pH assay (n=60)**					

**Controls**	**Control 1**	**Control 2**	**Control 3**		**Control 4**

Mean	3.6	7.0	10.0		11.5
Within-run SD	3.45 ± 0.1	7.18 ± 0.1	10.64 ± 0.1		11.50 ± 0.1
Within-run CV, %	3.6	1.2	1.0		1.0
Between-run SD	3.45 ± 0.2	7.18 ± 0.1	10.64 ± 0.1		11.50 ± 0.2
Total CV, %	4.5	2.0	1.1		1.4

**C) Precision results for Thermo Fisher oxidant assay (n=120)**					

**Controls**	**Control 1**	**High calibrator**		**Control 2**	

Mean, µg/mL	100	200		300	
Within-run SD, µg/mL	84.8 ± 2.1	199.3 ± 2.9		322.1 ± 3.8	
Within-run CV, %	2.4	1.5		1.2	
Between-run SD, µg/mL	84.8 ± 2.7	199.3 ± 3.6		322.1 ± 5.8	
Between-run CV, %	3.2	1.8		1.8	

**D) Precision results for micro Palm Abbe digital refractometer PA202 for SG (n=20)**					

**Controls**	**Control 1**	**High calibrator**		**Control 2**	

Mean, g/mL	1.014	1.025		1.031	
Within-run SD, g/mL	1.020 ± 0.0004	1.028 ± 0.0002		1.032 ± 0.0002	
Within-run CV, %	0.05	0.05		0.06	
Between-run SD, g/mL	1.015 ± 0.0005	1.020 ± 0.0004		1.032 ± 0.0004	
Between-run CV, %	0.05	0.05		0.06	

**E) Precision results for Mettler Toledo Seven Compact™ pH/Ion meter S220 (n=20)**					

**Controls**	**Control 1**	**Control 2**	**Control 3**		**Control 4**

Mean	3.6	7.0	10.0		11.5
Within-run SD, pH	3.50 ± 0.1	7.19 ± 0.1	10.66 ± 0.1		11.56 ± 0.1
Within-run CV, %	1.8	1.1	1.1		1.2
Between-run SD, pH	3.45 ± 0.2	7.18 ± 0.1	10.64 ± 0.1		11.50 ± 0.2
Total CV, %	2.0	1.5	1.6		1.5

The results were also analysed based on the reference interval to evaluate the results from the clinical point of view to summarise the overall outcome of different methods. Based on the SG reference interval 1.003–1.035, there was 81.9% method agreement between Thermo Fisher chemical SG method on Beckman–Coulter AU5810 chemistry analyser and SG refractometer method. 66.1% method agreement between SG refractometer method and dipstick. 64.7% method agreement between Beckman–Coulter AU5810 chemistry analyser, SG methods and SG dipstick (Table 3).

**Table 3:** SG and pH methods comparison and agreement based on reference interval.

SG method comparison based on Reference Interval (RI) 1.003–1.035	SG (Beckman–Coulter AU5810 chemistry analyser,)	SG (Refractometer)	SG TECO™ Drug Adulteration Test Strip (dipstick)
Total no tested	204	204	204
Number of results within the reference interval	197	173	129
Number of results below RI	6	19	52
Number of results above RI	1	12	23
SG agreement between Thermo Fisher chemical method and the refractometer		167 out of 204 (81.9%)	
SG agreement between the refractometer and the TECO™ Drug Adulteration Test Strip (dipstick)		135 out of 204 (66.1%)	
SG agreement between Thermo Fisher chemical method and the TECO™ Drug Adulteration Test Strip (dipstick)		132 out of 204 (64.7 %)	

**pH methods comparison based on reference interval (RI) 4.5 – 8.0**	**pH** **(Beckman–Coulter AU5810 chemistry analyser)**	**pH** **(pH Meter)**	**pH** **TECO™ Drug Adulteration Test Strip (dipstick)**

Total no tested	210	210	210
Number of results below the reference interval	1	1	5
Number of results above the reference interval	84	128	102
pH method agreement between Thermo Fisher chemical method and pH meter method		156 out of 210 (74.3%)	
pH method agreement between pH meter and TECO™ Drug Adulteration Test Strip (dipstick)		180 out of 210 (85.7%)	
pH method agreement between Thermo Fisher chemical method and TECO™ Drug Adulteration Test Strip (dipstick)		171 out of 210 (81.4%)	

Based on the reference interval of pH 4.5–8.0, there was 74.3% method agreement between Thermo Fisher chemical method on Beckman–Coulter AU5810 chemistry analyser and pH meter method. A total of 85.7% method agrees between pH meter method and dipstick. A total of 81.4% method agrees between pH method agreement between Thermo Fisher chemical method on Beckman–Coulter AU5810 chemistry analyser and dipstick (Table 3).

Regarding the interference study, the results for serial dilutions for pyridinium chlorochromate, laboratory-grade sodium nitrite, nitrite certified reference material, sodium nitrate and sodium hypochlorite (Bleach) using oxidant assay and dipstick on day 1 and day 17 are summarised in Table 4. The pyridinium chlorochromate lowest concentration (25 µg/mL) was negative on day 17. Sodium hypochlorite concentrations deteriorated over time due to stability decrease [11] The Techo dipstick gives semiquantitative results for nitrite, pyridinium chlorochromate and sodium hypochlorite.

**Table 4:** Serial dilutions results for pyridinium chlorochromate, laboratory-grade sodium nitrite, certified reference material of nitrite and sodium hypochlorite (bleach) using oxidant assay and UrineCheck 7 Drug Adulterant Test Strip.

Pyridinium chlorochromate	Oxidant assay, µg/mL				TECO™ Drug Adulteration Test Strip – pyridinium chlorochromate (PC)		
	Day 1		Day 17		Day 1	Day 17	Creatinine, mmol/L
PC 400, µg/mL	674		537		Positive	Positive	100
PC 200, µg/mL	352		281		Positive	Positive	100
PC 100, µg/mL	185		140		Positive	Positive	100
PC 50, µg/mL	92		74		Positive	Positive	100
PC 25, µg/mL	47		32		Positive	Negative	100
Blank urine	0		0		0	0	100

**Sodium nitrite (laboratory-grade)**	**Oxidant assay, µg/mL**				**TECO™ Drug Adulteration Test Strip – nitrite**		
	**Day 1**		**Day 17**		**Day 1**	**Day 17**	**Creatinine, mmol/L**

Nitrite 400, µg/mL	328		317		Positive (>15 mg/dL or >150 µg/mL)		100
Nitrite 200, µg/mL	146		141		Positive (>15 mg/dL or >150 µg/mL)		100
Nitrite 100, µg/mL	59		63		Positive (>15 mg/dL or >150 µg/mL)		100
Nitrite 50, µg/mL	28		27		Positive (0.5–5 mg/dL or 5–50 µg/mL)		100
Nitrite 25, µg/mL	12		11		Positive (0.5–5 mg/dL or 5–50 µg/mL)		100
Blank urine	0		0		0		100

**Nitrite (Certified Reference Material)**	**Oxidant assay, µg/mL**				**TECO™ Drug Adulteration Test Strip − nitrite**		
	**Day 1**		**Day 17**		**Day 1**	**Day 17**	**Creatinine, mmol/L**

Nitrite 1,000, µg/mL	1,157		1,156		Positive (>15 mg/dL or >150 µg/mL)		100
Nitrite 500, µg/mL	542		543		Positive (>15 mg/dL or >150 µg/mL)		100
Nitrite 250, µg/mL	259		257		Positive (>15 mg/dL or >150 µg/mL)		100
Nitrite 125, µg/mL	122		123		Positive (0.5–5 mg/dL or 5–50 µg/mL)		100
Nitrite 62.5, µg/mL	58		59		Positive (0.5–5 mg/dL or 5–50 µg/mL)		100
Blank urine	0		0		0		100

**Sodium nitrate (laboratory-grade)**	**Oxidant assay, µg/mL**			**TECO™ Drug Adulteration Test Strip** − **nitrate**			
	**Day 1**	**Day 17**		**Day 1**		**Day 17**	**Creatinine, mmol/L**

Nitrate 400, µg/mL	0			0			100
Nitrate 200, µg/mL	0			0			100
Nitrate 100, µg/mL	0			0			100
Nitrate 50, µg/mL	0			0			100
Nitrate 25, µg/mL	0			0			100
Blank urine	0			0			100

**Sodium hypochlorite (bleach)**	**Oxidant assay, µg/mL**			**TECO™ Drug Adulteration Test Strip (Sodium hypochlorite)**			
	**Day 1**		**Day 17**	**Day 1**		**Day 17**	**Creatinine, mmol/L**

Bleach 4%	1,058		135	Positive		Positive	100
Bleach 2%	455		13	Positive		Negative	100
Bleach 1%	149		2	Positive		Negative	100
Bleach 0.5%	42		0.7	Positive		Negative	100
Blank urine	0		0	Negative		Negative	100

## Discussion

SG is a measurement of the density of a liquid compared to the density of water. It measures the concentration of dissolved particles in the sample. Decreased SG values may be due to excessive fluid intake, renal failure, diabetes insipidus, as well as a variety of other factors. Increased SG values could be due to dehydration, renal dysfunction, and other medical factors including increased antidiuretic hormone secretion, which can be due to stress, trauma and some drugs.

Refractometry measures the refractive index, which is related to the total mass of solutes present in the urine. High molecular weight substances such as glucose, protein or radiographic contrast agents will have a greater effect on the SG. In contrast, reagent strips measure ionic strength and are not affected by protein, glucose, or contrast agents. Osmolality, which measures SG indirectly is affected by glucose but not by contrast agents [12]. It was found that pathological urines had a significantly poorer correlation between SG and osmolality than “clean” urines. The variables that affect the correlation are pH, ketones, bilirubin, urobilinogen, glucose, and protein for the dipstick method and ketones, bilirubin and haemoglobin for the refractometry method [13].

The distributions of the data points for the dipstick method in Deming regression seem to be in some parts distributed in horizontal lines is that the dipstick method used has a limited measuring quantitative range for pH and SG. Also, does not report decimals for pH which is not the case with the other two methods. This is a limitation of the dipstick method.

In a published study, SG was measured using Clinitek dipstick, refractometer and osmometer. The SG values of the refractometer and the osmometer had a good linear correlation. The tested Clinitek dipstick method was unreliable for the determination of SG, even after correction for pH, as recommended by the manufacturer [14]. But another study found that there is an agreement between SG dipstick method and the refractometer [15]. Such conflict can be explained because another study found that there were varying degrees of differences were seen in the SG measurements among the different refractometers. These differences were refractometer-dependent, and the results from one instrument could affect clinical decisions [16].

The refractive index of a solution changes based on the sum of all of the dissolved solids in that solution. The relationship between refractive index and urine SG or protein concentration is not the same for all refractometers, nor is the methodology of making the measurements [17].

The pH level determines the acidity or alkalinity of the sample. Although some biomedical conditions affect urine pH, the physiological range cut-off is 4.5–9.0. Urine specimen pH may be elevated up to 9.5 due to poor storage conditions such as elevated temperature. Ascorbate negatively interfered with haemoglobin, glucose and nitrite measurements. Acetylsalicylic acid lowered pH, the effect being greatest when protein was absent [18].

One of the major factors affecting urine pH is the food that a person consumes. Acidic foods include grains, fish, sodas, high-protein foods and sugary foods yield acidic urine.

Alkaline foods include nuts, vegetables and most fruits yield alkaline urine. If a person has a high urine pH, meaning that it is more alkaline, it might signal a medical condition such as kidney stones, urinary tract infections and kidney-related disorders. A person can also have a higher urine pH due to prolonged vomiting. This rids the body of stomach acid, which can make body fluids more basic.

Acidic urine can also create an environment where kidney stones can form. If a person has low urine pH, meaning that it is more acidic, it might indicate a medical condition such as diabetic ketoacidosis, which is a complication of diabetes, diarrhoea and starvation. Taking certain medications can also make a person’s urine pH more basic or acidic. Certain medications should be stopped the night or morning of a urinalysis unless a doctor wants to determine a person’s urine pH while they are taking them.

If any of the SG and pH urine parameters are outside the specified range, there is reason to believe that the urine sample has been adulterated.

Several oxidising adulterants are being sold with a claim to clear all positive drug test results.

For example, oxidizing adulterants are nitrite (Klear™), chromate (Urine Luck™), iodine, bleach and horseradish peroxidase/H_2_O_2_ (Stealth™). When added to urine, there is no significant change to the appearance, pH, SG or creatinine concentration. Marijuana samples adulterated with oxidants can produce a positive result, during an initial screening by immunoassay, notably the marijuana metabolite tetrahydrocannabinol (THC). However, they can not be confirmed by GC-MS or LC-MS [19], [20].

The general oxidant-detect test can be performed on automated clinical chemistry analysers to detect oxidants. The method is based on the reaction between the substrate tetramethylbenzidine (TMB) and the oxidant in the sample producing colour that can be measured at 660 nm. Some oxidants such as nitrite may be generated in the human body and excreted into urine through enzymatic oxidation by nitric oxide synthase (NOS). However, most nitrite formed is oxidised to nitrate. Therefore, nitrate concentration in human urine from NOS activity is much greater than the nitrite concentration. A study was conducted with healthy volunteers and reported an average urine concentration of 61 μg/mL for nitrate and 0.2 μg/mL for nitrite [21]. Patients with urinary tract infection or pathological conditions may have urine nitrite as high as 100–150 μg/mL. Urine samples to which Klear™ was added as a nitrite source were found to contain between 1,900 and 15,000 μg/mL nitrite. Therefore, a urinary nitrite concentration of 200 μg/mL or greater is a scientifically valid and forensically defensible proof of adulteration of the specimen with a nitrite-containing substance. Chromate is also present in the human body at very low concentration. The normal urinary chromium concentrations range from 0.04–1.0 μg/mL [21], [22].

## Conclusions

SG method agreement of the Thermo Fisher chemical method was closer to the refractometer method more than the TECO™ Drug Adulteration Test Strip. pH method agreement of the Thermo Fisher chemical method was closer to the TECO™ Drug Adulteration Test Strip more than the pH meter method.

Based on the findings and for consistency, accuracy and faster turn-around time, it is recommended to adopt the automated chemical methods to test for creatinine, oxidant assay, pH and SG for sample validity testing. If a urine oxidant assay is negative and the pH and/or SG results are not within their reference intervals, it is recommended to test the suspected urine using Axiom assay™ from Axiom Diagnostics incorporated to test for synthetic urines.
